# Patterns and Determinants of Change in Cortisol Levels and Thyroid Function as a Function of Cardiac Risk in Children Undergoing Cardiac Surgery

**DOI:** 10.1155/2022/6730666

**Published:** 2022-02-22

**Authors:** Khouloud Abdulrahman Al-Sofyani, Mohammed Shahab Uddin, Ebtehal Ahmed Qulisy, Osman Osama Al-Radi

**Affiliations:** ^1^Department of Pediatric, Faculty of Medicine, King Abdulaziz University, Jeddah, Saudi Arabia; ^2^Department of Pediatric, Imam Abdurahman Bin Faisal Hospital, Dammam, Saudi Arabia; ^3^Department of Surgery, Cardiac Surgery Unit, Faculty of Medicine, King Abdulaziz University, Jeddah, Saudi Arabia

## Abstract

**Background:**

Children undergoing cardiac surgery with cardiopulmonary bypass (CPB) are exposed to the risk of hormonal imbalances resulting from acute stress, which may eventually result in high postoperative mortality and morbidity.

**Objective:**

We assessed adrenal and thyroid hormonal changes and their determinants following cardiac surgery in children and explored their prognostic value in predicting cardiac outcomes. *Study Design and Methods*. A prospective cohort study was conducted at King Abdulaziz University Hospital (KAUH), between 2017 and 2018. The study involved 46 children aged 14 years or younger who underwent elective cardiac surgery with cardiopulmonary bypass. Serum levels of cortisol, TSH, fT3, and fT4 were measured preoperatively and 24, 48, and 72 hours after surgery. The cardiac risk was assessed using the risk adjustment for congenital heart surgery (RACHS) scale. A composite cardiovascular outcome was analyzed as a numerical variable and calculated as the number of cardiovascular events.

**Results:**

Overall, the changes in thyroid function parameters resulted in a U-shaped curve, while cortisol levels yielded a bell-shaped curve. The most significant changes occurred at 24 hours postop, including a decrease in mean TSH by 2.08 *μ*IU/L (*p* < 0.001), fT3 by 2.39 pmol/L (*p* < 0.001), and fT4 by 2.45 pmol/L (*p* < 0.001) and an increase in cortisol levels by 406.48 nmol/L (*p* < 0.001) with respect to the baseline. Cortisol concentration peaked higher and recovered slower among patients with high cardiac risk than their counterparts. Cardiovascular outcomes were independently predicted by the extent of the decline in fT4 and TSH at 48 and 72 hours postop, with reference to the baseline, and by the cortisol level at 24 h postop, independent of the baseline, besides the RACHS category.

**Conclusion:**

Cardiac surgery among children yields a high adrenocortical response and a high incidence of nonthyroidal illness syndrome, increasing cardiovascular risk. A preventive management strategy involves improving surgical techniques to minimize trauma-related stress.

## 1. Introduction

Stress caused by cardiac surgery induces acute disturbances in the organism homeostasis, most notably during the procedures requiring cardiopulmonary bypass (CBP), which may ultimately lead to high postoperative morbidity and mortality. These disturbances are explained by the severe inflammatory state resulting from prolonged surgery, triggering neurohormonal responses of the hypothalamic-pituitary axes, which in turn are responsible for generalized pathological states [1–3]. The most remarkable among these neurohormonal responses is the surge in cortisol levels and decreased thyroid function, resulting from hyperstimulation of the hypothalamic-pituitary-adrenal axis and suppression of the hypothalamic-pituitary-thyroid one, respectively [4, 5].

In children undergoing cardiac surgery, either high cortisol or low thyroid hormone levels in the postoperative period were shown to be associated with more complicated postoperative management, including a higher requirement of vasoactive and inotrope medications and more frequent need for mechanical ventilation (MV), leading to longer PICU (pediatric intensive care unit) stays [6]. In contrast, other reports support that the hyperstimulation of the hypothalamic-pituitary-adrenal axis and the consequent hypercortisolemia is a physiological response associated with the recovery process. The same data suggest that normal or low serum cortisol concentration in the early postoperative time is indicative of inadequate adaptation to stress and is associated with poor hemodynamic condition [7–9]. In such cases, hydrocortisone therapy was advocated and clinical applications showed improved hemodynamic profile and lowered the requirement of vasoactive-inotrope medications [10].

On the other hand, suppression of the hypothalamic-pituitary-thyroid axis results in transient hypothyroidism syndrome called nonthyroidal illness syndrome, which is characterized by a decrease in thyroid hormone levels, more markedly T3, in the absence of primary thyroid disease [11, 12]. However, uncertainty remains about whether nonthyroidal illness syndrome is an adaptive state that promotes recovery or a genuine organ failure indicating maladaptation of the organism to surgical stress [13, 14]. Consequently, the question regarding the usefulness of supplementation therapy in improving outcomes among children and neonates undergoing prolonged cardiac surgery has evolved but remains controversial [15, 16]. However, the evolution of these neuroendocrine disturbances over time and their role as a function of cardiac risk assessment remain unclear.

We hypothesized that significant differences in the extent of change and recovery time in cortisol and thyroid function might be observed between low-risk and high-risk categories of patients due to the difference between the two groups in the preoperative stress caused by the severity of the cardiac disease and the overall hemodynamic and systemic condition as well as the complexity of the surgical procedure. The present study was designed to probe this hypothesis and the patterns of change in thyroid function tests, including fT3, unbound thyroxine (fT4), and thyroid-stimulating hormone (TSH), and total cortisol levels were monitored for over three days postoperation. Further, all four parameters were analyzed as a function of the RACHS (risk adjustment for congenital heart surgery) category. Subsequently, other preoperative and intraoperative predictors of the neuroendocrine responses were analyzed. Furthermore, we analyzed the pre- and postoperative levels of fT3, fT4, TSH, and cortisol levels as cofactors of composite cardiac outcomes besides cardiac risk assessment.

## 2. Materials and Methods

### 2.1. Design and Setting

A prospective cohort study was conducted among the children who underwent cardiac surgery at the pediatric intensive care unit (PICU), King Abdulaziz University Hospital (KAUH), Jeddah, Saudi Arabia, between 2017 and 18. The study protocol was reviewed and ethically approved by the institutional review board of KAUH (Reference No. 351-14). All data were collected anonymously, respecting the confidentiality of the patients, and informed consent was taken from the parents.

### 2.2. Population and Sampling

Inclusion criteria applied for patients aged 14 years old or younger who underwent elective surgical repair with CPB for congenital heart disease. Children with a preexisting endocrine disease on hormonal replacement were excluded. Furthermore, children missing outcome data, notably preoperative and postoperative monitoring of thyroid function test and cortisol, were excluded. A convenience sampling was used to include all eligible patients.

### 2.3. Procedure

Serum concentrations of total cortisol (normal range: 138–636 nmol/L), thyroid-stimulating hormone (TSH) (normal range: 0.27-4 *μ*IU/mL), and thyroid hormone levels (free T3 (fT3) (normal range: 2.8–7 pmol/L) and free thyroxine (fT4) (normal range: 12–20 pmol/L)) were measured. Sera were extracted from blood samples collected with the help of arterial or central venous indwelling catheters. All parameters were measured during the preoperative time or on induction of anesthesia as baseline and 24, 48, and 72 hours postoperatively. Blood samples were drawn between 0400 hrs and noon from indwelling venous catheters.

### 2.4. Hormone Assays

fT3, TSH, and fT4 were measured using Roche electrochemiluminescence immunoassay, while monoclonal antibody ELISA measured total cortisol. This antibody has no cross-reactivity with dexamethasone and around 12.9% cross-reactivity with methylprednisolone.

### 2.5. Study Data and Variables

Study data comprised of four categories:

1. Demographic and baseline clinical data, including age, gender, medical and surgical history, preoperative medication, cardiac risk assessment (RACHS category), and the types of heart surgeries performed
2. Intraoperative data including CPB time, aorta cross-clamp time, and deep hypothermic circulatory arrest (DHCA) time
3. Postoperative data including whether chest was kept open postop, mechanical ventilation (MV) duration, PICU length of stay, postoperative hospital length of stay, need for urgent Cath, temporary pacing, cardiac function, inotrope or vasoactive drug requirement, cardiac complications (hypotension, low cardiac output syndrome (LCOS), and arrhythmia), and other complications (acute kidney injury (AKI), sepsis, pneumothorax, bleeding, and phrenic nerve palsy), besides glycemic control and mortality. The cardiac function was assessed by transthoracic echocardiogram combined with hemodynamic parameters, and LCOS was defined as any patient requiring inotropic support with low myocardial function as determined by ejection fraction (EF) less than 55%. Acute kidney injury (AKI) was defined according to the Kidney Disease Improving Global Outcomes (KDIGO) criteria [17]
4. Laboratory data including preoperative and 24 h, 48 h, and 72 h postoperative levels of cortisol, TSH, fT3, and fT4

A composite cardiac outcome (CCO) was defined as the occurrence of any of the following cardiac events in the postoperative time: the need for urgent Cath, temporary pacing, inotrope or vasoactive drug requirement, hypotension, LCOS, and arrhythmia. It was analyzed as a numerical variable indicating the number of cardiac events.

### 2.6. Statistical Methods

The data was entered, coded, and cleaned in Microsoft Office Excel Professional Edition 2013. Statistical analysis was performed with the Statistical Package for the Social Sciences version 21.0 for Windows (SPSS Inc., Chicago, IL, USA). Categorical variables are presented as frequency and percentage, while numerical variables are presented as the mean ± standard deviation (SD) for normally distributed and median (centile) for nonnormally distributed ones.

The paired *t*-test was used to analyze the changes in the thyroid function tests (TSH, T4, and T3) and cortisol levels at 24, 48, and 72 hours postop with respect to the preoperative time. Results are presented as the mean and mean pre-to-postop differences, with respective significance levels.

Repeated-Measure Analysis of Variance (RM-ANOVA) was carried out to analyze the effect of time and cardiac risk assessment (RACHS category ≥ 3 versus <3) and the interaction between the two factors on the variance in the outcome variables (TSH, T4, fT3, and cortisol); Wilk's Lambda and Pillai's Trace statistics were calculated, as appropriate, and the effect size was expressed by partial Eta squared. Where the effect of time was significant, pairwise comparison analyses were carried out to determine the time points entailing significant change with reference to the baseline (preoperative measurements).

We hypothesized that patients with higher cardiac risk would display an early postop adverse response in the studied hormones including a decrease in TSH, fT3, and or fT4 and an increase in cortisol levels with reference to the baseline. To test this hypothesis for each parameter, the participants were categorized into early responders and nonresponders according to the pattern of early postop response (at 24 h postop) for the given parameter. For TSH, fT3, and fT4, early responders were defined as the patients who had any decrease at 24 h postop with reference to the baseline. For cortisol, early responders were defined as patients who had >10% increase in cortisol level at 24 h postop with reference to the baseline. The association of cardiac risk assessment with early response was analyzed by comparing the percentage of early responders in RACHS category ≥ 3 versus <3 with respect to the parameter, using the chi-squared test.

Recovery of thyroid function tests was defined as the restoration of at least 90% of the baseline level of thyroid function, and the association of cardiac risk assessment with time to recover was analyzed by comparing the percentage recoveries at 48 h and 72 h and nonrecoveries in RACHS category ≥ 3 versus <3 with respect to the parameter, using the chi-squared test.

Stepwise linear regression was carried out to analyze the independent factors associated with the most significant change in TSH, T3, T4, and cortisol levels in the postoperative period. Results are presented as the odds ratio with 95% confidence interval (95% CI) and the correlation coefficient (*R*^2^).

Two multivariate models were built to analyze the prognostic value of the studied neuroendocrine parameters on the postoperative cardiac outcome (indicated by CCO), using stepwise linear regression. The first model, Model A, included raw values of preoperative and postoperative measurements of TSH, fT3, fT4, and cortisol. The second model, Model B, included baseline-to-postop changes in the four parameters calculated as Δ*X*_*T*_ = *X*_*T*_–*X*_*preop*_, where Δ*X*_*T*_ is the extent of change in the given parameter at the given postoperative time (24 h, 48 h, or 72 h), *X*_*T*_ is the raw value of the given parameter at the given postoperative time, and *X*_*preop*_ is the raw preoperative value of the same parameter. Both models included preoperative cardiac risk assessment, indicated by the RACHS category, as a cofactor. Results are presented as the odds ratio with 95% confidence interval (95% CI) and the correlation coefficient (*R*^2^).

A *p* value of <0.05 was considered to reject the null hypothesis.

## 3. Results

### 3.1. Participants' Demographic and Preoperative Characteristics

Forty-six children were included in the study where 25 (54.3%) were male, median age = 12 months (range 1 month, 14 years). Six (13.0%) patients had previous cardiac surgery. Preoperative assessments showed that 26 (56.5%) were categorized as RACHS ≥ 3. The majority of the participants were on angiotensin-converting enzyme inhibitors (73.9%) and/or digoxin (54.3%) during the preoperative period (Table 1). The types of heart surgeries performed in this study are presented in Supplementary Table 1.

### 3.2. Intraoperative Parameters and Postoperative Data

All patients required mechanical ventilation during surgery. The median time for the cardiopulmonary bypass (CPB) and aorta cross-clamp was 67 minutes and 46.5 minutes, respectively. Postoperative data showed that the sternal closure was delayed for 8 (17.4%) patients, and median mechanical ventilation duration, PICU, and hospital length of stays were 1.5, 5.5, and 8.5 days, respectively. Postoperative cardiac function was good in most patients (60.9%) and fair in the remaining. The most frequent postoperative complications were hypotension (37.0%), low cardiac output syndrome (LCOS, 37.0%), and acute kidney injury (AKI, 26.1%). Meanwhile, postoperative medications to the patients included inotropes (29 patients, 63.0%), antibiotics (21 patients, 45.7%), and milrinone (42 patients, 91.3%). All the patients (100%) were provided with anti-heart failure medications, while three (6.5%) patients were suggested L-thyroxine in their discharge medication (2 of them had Down syndrome). Only one (2.2%) mortality was reported among the patients during the study (Table 2).

### 3.3. Postoperative Changes in Thyroid Function and Cortisol Level

**Figure 1:**
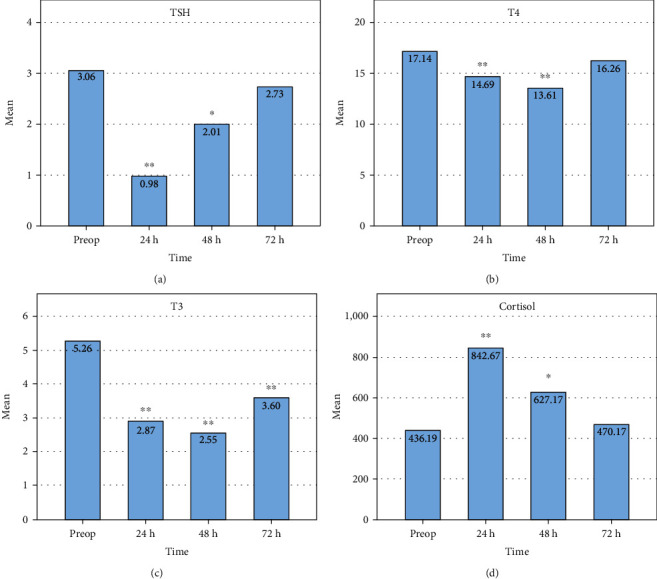
Changes in thyroid function and cortisol level in the 72-hour postoperative period. Bars represent the mean levels of (a) TSH, (b) fT4, (c) fT3, and (d) cortisol in the preoperative period and 24, 48, and 72 hours postop. Significance levels are calculated with reference to the levels observed in the preoperative period: ^∗∗^*p* < 0.01;  ^∗^*p* < 0.05.

Overall, the changes in thyroid function parameters (TSH, fT3, and fT4) resulted in a U-shaped curve when analyzed from the preoperative period to 72 hours postop, while cortisol levels yielded a bell-shaped curve with the apex at 24 h postop. The early postoperative time was marked by a significant decrease in TSH (by 2.08 *μ*IU/L (*p* < 0.001) then 1.05 *μ*IU/L (*p* = 0.037)), fT3 (by 2.39 pmol/L (*p* < 0.001) then 2.71 pmol/L (*p* < 0.001)), and fT4 (by 2.45 pmol/L (*p* < 0.001) then 3.53 pmol/L (*p* < 0.001)), at 24 h and 48 h, respectively, with reference to the baseline. On the other hand, cortisol levels increased by 406.48 nmol/L (*p* < 0.001) and 190.89 nmol/L (*p* = 0.025) at 24 h and 48 h postop, respectively, with respect to the baseline. At 72 hours postop, TSH, fT4, and cortisol levels were completely restored to the preoperative levels, while fT3 was partially restored but remained 1.67 pmol/L lower than the baseline (*p* < 0.001) (Figure 1).

**Figure 2:**
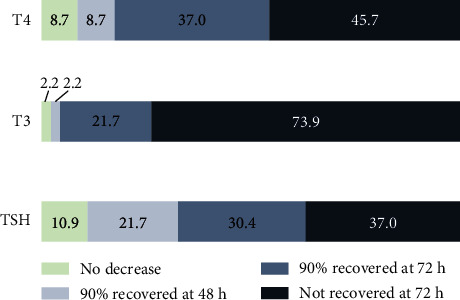
Time taken for the recovery of thyroid function tests following cardiac surgery among children. Among the participants, 10.9%, 2.2%, and 8.7% (first portion of the bars from the left) had no decrease in TSH, fT3, and fT4, respectively. Recovery rates are shown at 48 and 72 hours postop, as well as the percentage of participants who had no recovery.

Analyses of the time taken for the recovery of thyroid function showed that majority of the patients showed 90% recovery to the baseline TSH and fT4 levels at 72 hours postop, whereas only 23.7% of the patients showed 90% recovery of the baseline fT3 levels (Figure 2).

Bivariate correlation analysis showed a weak positive correlation between the changes in fT4 and fT3 at 24 hours (*r* = 0.323, *p* = 0.029), 48 hours (*r* = 0.430, *p* = 0.003), and 72 hours (*r* = 0.389, *p* = 0.007). Also, there was a weak positive correlation between the change in fT3 and TSH at 48 hours (*r* = 0.435, *p* = 0.003) and 72 hours (*r* = 0.495, *p* < 0.001). On the other hand, a weak negative correlation was found between the change in fT3 and cortisol at 48 hours post-intervention (*r* = −0.328, *p* = 0.026).

### 3.4. Impact of Cardiac Risk Category on Changes in Thyroid Function and Cortisol Levels

**Figure 3:**
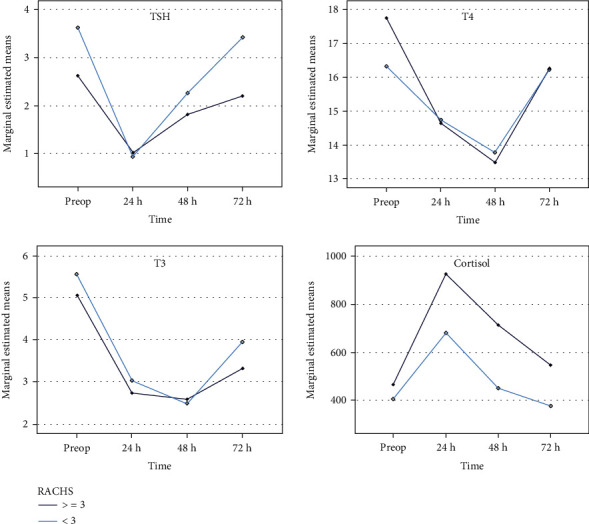
Changes in the thyroid function and cortisol levels over the early postoperative period (0-72 hours postop) following cardiac surgery with cardiac risk category score assessment (RACHS level).

No effect of the RACHS category (≥3 versus <3) or the interaction Time∗RACHS category was observed on TSH, fT3, or fT4; all show low partial Eta-squared values with no statistically significant result. However, there was a moderate effect of the RACHS category on the change in the cortisol level (partial Eta squared = 0.109, *p* = 0.027); this restoration of the cortisol levels was achieved earlier in the low-risk group (RACHS < 3) as compared with their counterparts (Table 3). Results and statistics of RM-ANOVA are depicted in Table 3, while the marginal estimated mean of all four parameters by RACHS category is depicted in Figure 3. Further, there was no statistically significant difference between RACHS categories ≥ 3 versus <3 regarding the percentage of early responders (decrease at 24 hours postop) or time needed to recover 90% of the baseline levels of TSH (*p* = 3.69 and 0.251), fT4 (*p* = 1.000 and 0.541), or fT3 (*p* = 1.000 and 0.547), respectively. Similarly, no significant difference was observed in the percentage of early responders for cortisol (increase > 10% at 24 hours postop, by reference to the baseline) between RACHS categories ≥ 3 and <3 (73.1% versus 50.0%, respectively; *p* = 0.105).

### 3.5. Predictors of the Change in TSH, fT3, fT4, and Cortisol Postcardiac Surgery

The most significant decrease in the TSH occurred 24 h postop, and it was predicted by DHCA time (OR = 0.67, *p* < 0.001) and cardiac risk category (RACHS < 3 : OR = 0.18, *p* = 0.049), explaining 29.6% of the variance of TSH in the first 24 hours postop. For both fT3 and fT4, a significant decrease occurred at 24 h postop to reach the lowest point at 48 h postop. The change in fT3 was predicted by age (OR = 1.03, *p* = 0.023) in the first 24 hours postop, explaining 11.4% of the variance of fT3, and by both AKI (OR = 0.02, *p* = 0.003) and age (OR = 10.3, *p* = 0.035) at 48 h postop, explaining 26.9% of the variance. The change in fT4 was predicted by bypass time (>70 min: OR = 0.29, *p* = 0.017) at 24 h and by gender (female: OR = 0.33, *p* = 0.031), explaining 12.4% and 10.4% of the variance, respectively. Regarding cortisol, the most significant increase occurred 24 h postop and was predicted by both bypass time (>70 min) and delayed sternal closure postop, and the two predictors explained 29.6% variance in the cortisol levels (Table 4).

### 3.6. Prediction of Composite Cardiac Outcome Using RACHS Category and Thyroid Function Tests and Cortisol Monitoring

Stepwise linear regression Model A showed RACHS category (OR = 1.95 (95%CI = 1.11‐3.45), *p* = 0.022) and 24 h postop cortisol concentration (OR = 1.08 (95%CI = 1.01‐1.17), *p* = 0.045) to be independently associated with the number of cardiac events, and this model explained 21.5% of the variance of the dependent variable (*R*^2^ = 0.215). It is important to note that the OR for preoperative cortisol is expressed for every 100 nmol/L increment in the cortisol level. Of the other variables excluded from the model, preoperative TSH was the only parameter approaching statistical significance (OR = 1.30, *p* = 0.051). Thus, the predictive equation for this model is expressed as follows:

(1) $$\begin{array}{c} \text{CCO}=0.13+0.67*\text{X}1+0.08*\text{X}2, \end{array}$$

where CCO is the composite cardiac outcome indicating the number of postop cardiac events among X1 (RACHS category (1–6)) and X2 (24 h postop cortisol concentration/100).

Stepwise linear regression Model B showed that the extent of change in T4 at 48 h postop (OR = 0.83 (95%CI = 0.75–0.93), *p* = 0.02) and TSH at 72 postop (OR = 0.88 (95%CI = 0.77–0.99), *p* = 0.034), with reference to the baseline, were independently associated with CCO in an inverse relationship. A decrease in the T4 and TSH levels predicts a likelihood of more cardiac events, while an increase predicts the likelihood of lesser cardiac events. The model explained 24.8% of the CCO variance, and the predictive equation is expressed as follows:

(2) $$\begin{array}{c} \text{CCO}=1.97-0.18*\Delta \text{T}44_{8\text{h}}-0.13*\Delta \text{TSH}7_{2\text{h}}. \end{array}$$

## 4. Discussion

### 4.1. Summary of Findings

This prospective study described the patterns, explored the determinants, and analyzed the prognostic value on cardiac outcome based on the neuroendocrine changes following pediatric cardiac surgery. The data showed a high incidence of nonthyroidal illness syndrome with complete recovery of TSH and fT4 and partial recovery of fT3 by 72 h postop. The extent of change of these parameters varied depending on the patient's age, gender, cardiac risk category, CBP, and DHCA time, which was evident with up to 30% variance depending on the parameter. On the other hand, the most significant increase in the cortisol levels occurred at 24 h postop, and it was predicted by CBP time and delayed postop chest closure, explaining ~30% variance. While no association was found between cardiac risk and thyroid function changes, cortisol concentration peaked higher and was likely to recover slower among the patients with high cardiac risk than their counterparts. The number of postoperative cardiac events was independently predicted by the extent of the decline in fT4 and TSH at 48 and 72 hours postop, with reference to the baseline, and the cortisol level at 24 h postop, independent of the baseline, besides the RACHS category.

### 4.2. Response of the Hypothalamic-Pituitary-Thyroid Axis in Pediatric Cardiac Surgery

The present study confirmed the high incidence of thyroid dysfunction in the patients following cardiac surgery with CBP, which was characterized by an early and significant decrease in TSH, fT3, and fT4 in nearly all patients, followed by complete recovery at 72 hours postop in the majority of the patients, except for fT3 levels which entailed the highest decline rate (98%) and the lowest recovery rate at 72 h postop (~26%). These findings are consistent with the literature showing a persistent and more common decline in fT3 following cardiac surgery, both among children and adults and both with and without CBP [18, 19]. The most suspected pathophysiological mechanism for thyroid dysfunction is the generalized inflammatory response caused due to the surgical insult, resulting in organ failure. Strong correlations were found between peak serum levels of proinflammatory cytokines and the low levels of circulating thyroid hormones [18, 20]. In the present study, data of proinflammatory markers was not collected; however, the significance of the RACHS category and the CBP and DHCA duration in predicting thyroid dysfunction may correlate with the extent and duration of the generalized inflammatory state. Another suggested mechanism was the transcutaneous absorption of iodine in surgical iodinated antiseptics, resulting in iodine-induced hypothyroidism. This mechanism, also known as the Wolff-Chaikoff effect, was mostly observed in delayed sternal closure as wounds of such patients often require irrigation with povidone-iodine solution [21–23].

The present study demonstrated that the extent of decline in the thyroid function is predictive of the number of postoperative cardiac events and suggests that an increase in thyroid function would have a cardioprotective effect. These findings favor the beneficial effect of thyroid hormone supplementation in the early postoperative phase to prevent a dramatic decline of thyroid function. In literature, the prognostic value of thyroid hormone decline following cardiac surgery has been well documented in the last few decades. Low TSH and thyroid hormones, notably free T3, were highly predictive of postoperative cardiac events and overall cardiovascular mortality [18].

It is well known that thyroid hormones have inotropic effects leading to improving contractile status, besides reducing systemic vascular resistance. These effects are mediated by several mechanisms, including the expression of adrenergic receptors and regulation of calcium handling proteins in myocytes and an increase in metabolic activity and oxygen consumption, resulting in the release of local vasodilators. Besides, thyroid hormones have a direct vasoactive effect on arteriolar smooth muscle, notably in coronary arteries [15, 20]. However, results from clinical trials regarding the therapeutic value of thyroid hormone supplementation are ambivalent and insufficient to come up with evidence-based recommendations. In adults undergoing cardiac surgery with CBP, several studies and trials demonstrated the beneficial effect of administering T3 on improving patients' hemodynamics and cardiac output, decreasing systemic vascular resistance, and reducing the incidence of postoperative cardiac complications such as arrhythmia and myocardial ischemia; all lead to lesser need for inotrope agents and diuretics, shorter postoperative hospital stay, and better survival [24–29]. In children, although the few studies conducted so far were limited in size and design, they showed an improved cardiac index, notably in children with longer CBP time, besides the reduced requirement of inotrope medication and time to negative fluid balance [19, 30–34]. However, scrupulous reviews have concluded that there is a lack of evidence regarding either a beneficial or harmful effect of thyroid hormone supplementation in pediatric cardiac surgery, and thus, no evidence-based recommendation can be issued to date [15, 16]. A recent meta-analysis (2020), which included 9 randomized, placebo-controlled studies with 711 pediatric patients, showed a small but significant effect of thyroid hormone supplementation on reducing inotrope requirement, with no significant benefit in the postoperative course parameters including MV duration, PICU stay, hospital stay, cardiac index, or mortality. However, a small but significant reduction of PICU and hospital stay was observed with higher doses of thyroid hormones [35]. As a consequence, prophylactic management of the patients should principally focus on improving the technical aspects of the surgical approach so as to reduce the extent and duration of the related stress. This could be achieved by reducing procedure time, CBP time, and DHCA time and promoting the use of less traumatic instruments and noniodinated antiseptics, notably in the case of prolonged procedures and delayed sternal closure.

### 4.3. Response of the Hypothalamic-Pituitary-Adrenal Axis in Pediatric Cardiac Surgery

The present study showed that the adrenocortical response occurred in the early postoperative time, and the cortisol peak was proportional to the complexity of the surgery and was predicted by longer bypass time and delayed sternal closure. The postoperative period following complex cardiac surgery is associated with considerable hemodynamic instability, which is a consequence of the generalized inflammation, potentiated by several other factors such as pulmonary hypertension, DHCA, congestive heart failure, and CBP. These factors explain the hyperactivation of the hypothalamic-pituitary-adrenal axis leading to the secretion of cortisol, which is essential for both suppressing inflammation and enhancing vascular responsiveness to catecholamines [36]. Thus, this increased adrenocortical activity is considered a physiological response to stress, and its absence marks secondary adrenal insufficiency characterized by an increased ACTH/cortisol ratio and is associated with poorer outcomes [7–9]. In our study, hypercortisolemia was likely to be related to the grade of surgery invasiveness, controlling the overall amount of stress as it was directly associated with RACHS category, CBP time, and delayed sternal closure, all being potential factors of generalized inflammation. A systematic review showed that high-grade surgical procedures induced higher cortisol response with peaks detected intraoperatively or within the first 24 h postop [37].

Nonetheless, elevated postoperative cortisol levels following complex cardiac surgery are not always a result of optimal adrenal response as recent studies have shown that other abnormalities such as impaired metabolism, clearance of cortisol, or sensitivity of target tissues to glucocorticoid also lead to cortisol hyperproduction [36, 38]. These findings reject the relevance of empirical corticosteroid therapy, restricting its use for patients who have inadequate endogenous cortisol production due to documented adrenal insufficiency [15, 39]. However, no evidence-based recommendation has been issued to date.

Independent of the preoperative levels, elevated cortisol concentration at 24 h postop, combined with the RACHS category, was predictive for the number of cardiac events in the present study. An elevated cortisol level may indicate the invasiveness grade of the surgery and proportional level of inflammatory response. High cortisol levels in the postoperative period were shown to be associated with a higher requirement of vasoactive and inotrope drugs and more frequent mechanical ventilation (MV), requiring longer PICU stays [6]. In children with adrenal insufficiency, as indicated by the negative ACTH stimulation test, the postoperative outcomes were marked by a higher inotrope score, longer duration of mechanical ventilation, longer PICU stays, and low urine output than their counterparts [40]. These observations underline the importance of close monitoring and screening for adrenal insufficiency to enable better postoperative management and reduce morbidity and mortality.

### 4.4. Limitations

The present study is limited by the small sample size, resulting in unequal distribution of the patients across the RACHS category as 44 out of the 46 patients were category 2 or 3, which may not be significantly distinct from each other in terms of cardiac risk. Further, in the absence of data pertaining to proinflammatory markers, there is a gap in our understanding regarding the predictive value of the inflammatory response for the change in the cortisol levels or thyroid function tests. Another parameter to be taken into consideration for the generalization of these findings is the fact that most surgeries in our cohort required relatively short CPB time.

## 5. Conclusion

The invasiveness of cardiac surgery among children predicts the occurrence of hypercortisolemia and nonthyroidal illness syndrome, which are independently associated with poor cardiovascular outcomes. Scrupulous preoperative assessment enables anticipation of the complexity of the procedure by adapting the postoperative management to mitigate the odd risks. The early preoperative period is also critical to monitor the thyroid and adrenal functions for timely detection and management of any disturbance. The prevention of stress-related complications should rely on optimizing the technical parameters of surgery, which may include reducing procedure, CBP, and DHCA time, as well as using topical antiseptics during prolonged procedures and delayed sternal closure. Further research is warranted to explore the efficacy of such preventive measures, notably among children in the RACHS 3+ category.

## Figures and Tables

**Table 1:** Participants' demographic and preoperative characteristics (*N* = 46).

Parameter	Category	Frequency	Percentage
*Demographic characteristics*			
Gender	Male	25	54.3
	Female	21	45.7

Age (months)	Median (range)	12.0 (1.0-168.0)	—

*Medical and surgical history*			
Down syndrome	Yes	2	4.3
Past cardiac surgery	Yes	6	13.0

*Preoperative data*			
RACHS category	2	20	43.5
	3	22	47.8
	4	2	4.3
	5	1	2.2
	6	1	2.2

RACHS category	<3	20	43.5
	≥3	26	56.5

Preoperative medication	Captopril	34	73.9
	Digoxin	25	54.3
	Steroids	1	2.2

Results are frequencies/percentage except where otherwise specified.

**Table 2:** Intraoperative and postoperative characteristics (*N* = 46).

Parameter	Category	Frequency	Percentage
*Intraoperative data*			
CP bypass time	≤70 min	25	54.3
	>70 min	21	45.7

Aorta cross-clamp time (min)	Median (range)	50 (15-157)	—

DHCA	No	42	91.3
	Yes	4	8.7

*Postoperative data and complications*			
Chest kept open postop	Yes	8	17.4
MV duration (days)	Median (range)	1.5 (0.0-20.0)	—
PICU LOS (days)	Median (range)	5.5 (1.0-40.0)	—
Hospital LOS (days)	Median (range)	8.5 (1.0-60.0)	—
Urgent Cath	Yes	5	10.9
Temporary pacing	Yes	2	4.3

Postop heart function	Fair	18	39.1
	Good	28	60.9

Postop medications	Inotropes	29	63.0
	Antibiotics	21	45.7
	Steroids	2	4.3
	Milrinone	42	91.3

Complications			
AKI	Yes	12	26.1
Hypotension	Yes	17	37.0
LCOS	Yes	17	37.0
Arrhythmia	Yes	8	17.4
Sepsis	Yes	6	13.0
Pneumothorax	Yes	4	8.7
Reexploration for bleeding	Yes	7	15.2
Phrenic nerve palsy	Yes	1	2.2

Composite cardiac outcome	None	4	8.7
	1	10	21.7
	2	12	26.1
	3	4	8.7
	4	9	19.6
	5	4	8.7
	6	3	6.5

AKI stage	Stage I	8	17.4
	Stage II	4	8.7

Glycemic control	120-140 mg	31	67.4
	140-180 mg	15	32.6

Discharge medication			
Antiheart failure	Yes	46	100.0
L-Thyroxine	Yes	3	6.5

Results are frequencies/percentage except where otherwise specified. DHCA: deep hypothermic circulatory arrest; CP: cardiopulmonary; AKI: acute kidney injury; LCOS: low cardiac output syndrome; PICU: pediatric intensive care unit; MV: mechanical ventilation.

**Table 3:** Effect of cardiac risk category on changes in thyroid function and cortisol levels (Repeated-Measure Analysis of Variance (RM-ANOVA)).

Outcome	Factor	Statistics^§^	*p* value	Partial Eta squared	Comment (effect size)
TSH	Time	0.468	<0.001^∗^	0.468	Very large effect
	Time × RACHS	0.073	0.357	0.073	No effect
	Between-subjects	—	0.100	0.060	No effect

fT4	Time	0.466	<0.001^∗^	0.466	Very large effect
	Time × RACHS	0.051	0.524	0.051	No effect
	Between-subjects	—	0.824	0.001	No effect

fT3	Time	0.739	<0.001^∗^	0.739	Extremely large effect
	Time × RACHS	0.064	0.419	0.064	No effect
	Between-subjects	—	0.339	0.021	No effect

Cortisol	Time	0.717	0.003^∗^	0.283	Large effect
	Time × RACHS	0.955	0.595	0.045	No effect
	Between-subjects	—	0.027^∗^	0.109	Moderate effect

^§^Statistics used was Wilk's Lambda if both Levene's test of errors' variance equality and Box's test of equality of covariance matrices are verified (sig.>0.05 and > 0.01, respectively); otherwise, if either assumption is violated, then Pillai's Trace statistics was used. Effect size is assumed to be large if partial Eta squared > 0.14.

**Table 4:** Predictors of the change in TSH, T3, T4, and cortisol postcardiac surgery (stepwise linear regression).

Predicted parameter (time)	Predictor	Level	OR	95% CI	*p* value	*R* ^2^
TSH (24 h)	DHCA time	(min)	0.67	0.55-0.82	<0.001^∗^	0.296
	RACHS	<3	0.18	0.03-0.99	0.049^∗^	

fT3 (24 h)	Bypass time	(>70 min)	0.29	0.11-0.80	0.017^∗^	0.124

fT3 (48 h)	Gender	Female	0.33	0.12-0.90	0.031^∗^	0.104

fT4 (24 h)	Age	(Month)	1.03	1.00-1.06	0.023^∗^	0.114

fT4 (48 h)	AKI	Yes	0.04	0.00-0.53	0.016^∗^	0.269
	Age	(Month)	1.03	1.00-1.05	0.035^∗^	

Cortisol (24 h)	Bypass time	(>70 min)	[499.66]	[162.00]-[837.32]	0.005^∗^	0.296
	Delayed sternal closure	Yes	[609.65]	[170.79]-[837.32]	0.008^∗^	

AKI: acute kidney injury; OR: odds ratio; CI: confidence interval; DHCA: deep hypothermic circulatory arrest time; values between square brackets are unstandardized *B* values of the regression coefficient, as the corresponding OR values are very high. Variables included in the stepwise model are deep hypothermic circulatory arrest time (min), RACHS category, chest kept open postop (yes versus no), gender, age (months), bypass time (≤70 min versus >70 min), and acute kidney injury.

## Data Availability

The data used to support the findings of this study are included within the article.

## Abbreviations

CPB: Cardiopulmonary bypass
RACHS: Risk adjustment for congenital heart surgery
PICU: Pediatric intensive care unit
MV: Mechanical ventilation
DHCA: Deep hypothermic circulatory arrest
CCO: Composite cardiac outcome
LCOS: Low cardiac output syndrome
AKI: Acute kidney injury.

## Supplementary Materials

Supplementary Materials Supplementary Table 1: the types of heart surgeries performed in the study are listed below.
