# Supplementary material for: Patterns and Determinants of Change in Cortisol Levels and Thyroid Function as a Function of Cardiac Risk in Children Undergoing Cardiac Surgery
Source: Int J Pediatr. 2022 Feb 22;2022:6730666. doi: 10.1155/2022/6730666 (PMC8888083; doi:10.1155/2022/6730666)
Supplement: Supplementary Materials — Supplementary Table 1: the types of heart surgeries performed in the study are listed below. [file 6730666.f1.doc]

**Supplementary table 1:** The types of heart surgeries performed in the study are listed below.

| **Type of surgery** | **No of patients** |
| --- | --- |
| VSD closure | 8 |
| Arterial switch operation, VSD closure | 1 |
| Atrioventricular canal repair | 4 |
| Arterial switch operation | 5 |
| Coarctation repair | 2 |
| Subaortic membrane correction | 1 |
| Aortopulmonary window repair | 1 |
| Norwood with Sano Shunt | 1 |
| TOF repair | 3 |
| Aortoplasty | 1 |
| Pulmonary artery banding, BT shunt | 1 |
| Cavopulmonary shunt | 5 |
| Truncus repair | 2 |
| Bidirectional Glenn | 1 |
| Aortic arch repair, VSD closure | 1 |
| Pulmonary valve replacement | 1 |
| Replacement of extra cardiac conduit RV - PA | 1 |
| ASD, VSD closure | 1 |
| AVC repair, ASD closure, Mitral valve plasty | 1 |
| Arterial switch operation, Pulmonary artery banding | 1 |
| Mitral valve repair | 1 |
| ASD closure, MV replacement | 1 |
| Pulmonary artery banding | 1 |
| RVOT stent | 1 |

VSD- Ventricular septal defect; TOF- Tetralogy of Fallot; BT- Blalock-Taussig; MV- Mitral valve; RVOT- Right ventricular outflow tract; ASD- Atrial septal defect; AVC- Atrioventricular canal defect
